# Influence of short-term macronutrient deprivation in maize on photosynthetic characteristics, transpiration and pigment content

**DOI:** 10.1038/s41598-019-50579-1

**Published:** 2019-10-02

**Authors:** Krzysztof Sitko, Żaneta Gieroń, Michał Szopiński, Paulina Zieleźnik-Rusinowska, Szymon Rusinowski, Marta Pogrzeba, Agata Daszkowska-Golec, Hazem M. Kalaji, Eugeniusz Małkowski

**Affiliations:** 1 Department of Plant Physiology, University of Silesia in Katowice, Katowice, Poland; 2 Institute for Ecology of Industrial Areas, Katowice, Poland; 3 Department of Genetics, University of Silesia in Katowice, Katowice, Poland; 4 Department of Plant Physiology, Warsaw University of Life Sciences (SGGW), Warsaw, Poland

**Keywords:** Photosynthesis, Plant physiology, Plant stress responses

## Abstract

The aim of the research was to compare the impact of short-term deprivation of selected macronutrients (Ca, K, Mg and P) on the photosynthetic characteristics, transpiration and pigment content in maize. The strongest inhibition of photosynthesis was caused by a deprivation of Mg, which was visible as a decrease in the photosynthetic and transpiration rates, stomatal conductance, photosystem II (PSII) performance, chlorophyll and flavonol content with a simultaneously increased content of anthocyanins. In the K-deprived plants, a decrease in the photosynthetic rate was observed. However, the transpiration rate and stomatal conductance did not differ significantly compared with the control. In the K-deprived plants, a decrease in chlorophyll and an increase in the anthocyanin content were also observed. We showed that Ca starvation resulted in a decrease in the photosynthetic and transpiration rates, stomatal conductance and PSII performance, while the pigment content was not significantly different compared with the control. In the case of P-deprived plants, we observed a decrease in the photosynthetic and transpiration rates. Interestingly, the inhibition of stomatal conductance was the strongest in the P-deprived plants compared with all of the investigated elements. However, the performance of PSII was not significantly affected by P starvation compared with the control. Our results present for the first time a comprehensive analysis of the effect of short-term macronutrient deprivation on photosynthesis and transpiration in maize plants.

## Introduction

The life cycle of plants is affected by numerous abiotic factors and among them the mineral nutrients that are taken up by plant roots play a crucial role. Mineral nutrients are usually classified as macronutrients or micronutrients based on their concentration in plant tissues. The appropriate content of macro and micronutrients is required for the normal growth and development in plants, because a deprivation of macronutrients such as phosphorus, magnesium, potassium or calcium strongly affects plant metabolism^1–3^. Photosynthesis, which consists of two phases – light and dark reactions, is the key process in plant metabolism^4^. The research that has been conducted over the years has shown that photosynthesis is dependent on mineral nutrition due to the fact that many nutrients are involved in the electron transport chain^5^. Phosphorus is a nutrient that is necessary for the synthesis of ATP and other phosphorylated metabolites. Furthermore, a P deprivation causes a decrease in stomatal conductance and the transpiration rate, which are also associated with the photosynthetic rate. It has also been indicated that a low P content caused a reduction in the maximum quantum yield of PSII, which seemed to be the result of a reduced chlorophyll synthesis due to a P deprivation^6^. In addition, a deprivation of this element causes the active reaction centres to close, which reduces the electron transport from PSII to PSI^7–9^. Another important nutrient is magnesium, which is the activator of more than 300 enzymes (e.g. RNA polymerases, ATPases, protein kinases, phosphatases, glutathione synthase and carboxylases)^10^. Due to the central position of Mg in the chlorophyll molecule, it participates in the light reactions in the thylakoid membranes and promotes electron transport, which results in the generation of the H^+^ ion gradient across the thylakoid membrane. Under an Mg deprivation, the thylakoid membranes are disorganized, thus disabling the electron transport in PSII^2,11^. Mg promotes photochemical quenching by having a positive effect on photosynthesis^12^. Another macronutrient that plays an important role in photosynthesis is potassium. The research that has been conducted so far showed that a K deprivation leads to a reduction in photosynthetic efficiency due to a decrease in the photoprotection mechanisms, decrease in chlorophyll content, the degradation of chloroplast structure as well as changes in the Rubisco activity^13–16^. The decrease in the photosynthetic rate and stomatal conductance that is caused by a K deprivation has also been described by many authors^17–20^. Nevertheless, to the best of our knowledge, there have been no reports on the exact mechanism of the effect of a potassium deprivation on photosynthesis^16,21,22^. Another function of K is the regulation of stomatal movement, which is dependent on the K^+^ content. When potassium ions are transferred from the cells into the apoplast, the stomata close and transpiration is inhibited. Therefore, in conditions in which the K content is appropriate, higher values of parameters such as the water content in leaves, the osmotic potential, transpiration and stomatal conductance have been observed compared to plants that were under potassium deficient conditions^23,24^. Furthermore, the degree of stomatal opening, in addition to its direct impact on the water relations of plants, determines the CO_2_ uptake and thus indirectly has an influence on the photosynthesis rate^25–27^. In the case of calcium, which is another essential plant macronutrient, it is worth stressing its major role as an intracellular messenger in cytosol and thus its participation in the stress signal transduction in plant cells^2,28,29^. It also was found that the chloroplasts are part of the cellular Ca^2+^ network and that they contribute to the cytosolic Ca^2+^ signalling. Moreover, Ca^2+^ is a structural component of PSII and takes place in photosynthetic water oxidation, because this process, which occurs in PSII, requires a metal cluster that contains a calcium ion^29^. In addition, calcium has also been shown to improve photosynthetic efficiency and stomatal conductance in plants that had been treated with acid precipitation, as well as heat stress^30,31^, which is associated with an increased Rubisco activity in plants that had been treated with CaCl_2_^32,33^.

The aim of the research was to compare the impact of short-term deprivation of selected macronutrients (Ca, K, Mg and P) on the yield of the photosynthetic apparatus, transpiration and pigment content in maize. We used non-destructive methods for the measurements and portable devices with the aim of determining the stress markers that are caused by deprivation of the selected macronutrients. A simultaneous comparison of the effect of Ca, K, Mg and P deprivation on the chlorophyll *a* fluorescence, photosynthetic and transpiration rates, stomatal conductance and chlorophyll, anthocyanin and flavonol content was performed on maize for the first time. The removal of elements did not caused their deficiencies and decrease of growth. Thus, physiological processes investigated in the current study can be used as the early markers of selected macronutrient deprivation.

## Results

### Plant growth parameters and accumulation of elements

The macronutrient deprivation did not caused any significant changes in investigated growth parameters compared with the control (Table 1). However, each macronutrient deprivation was detected as significant decrease of its concentration in shoots, 4^th^ leaves and roots compared to the control (Table 1).

**Table 1:** Plant growth parameters and accumulation of elements in organs.

	control	Deprivation				
		Ca	K	Mg	P	
**Plant growth parameters**						
FW (g)	shoot	2.73 ± 0.30 a	2.50 ± 0.35 a	2.43 ± 0.22 a	3.30 ± 0.43 a	2.72 ± 0.29 a
	4^th^ leaf	0.65 ± 0.06 a	0.53 ± 0.03 a	0.50 ± 0.04 a	0.64 ± 0.06 a	0.58 ± 0.06 a
	roots	1.05 ± 0.11 a	0.82 ± 0.08 a	0.80 ± 0.08 a	1.10 ± 0.11 a	0.94 ± 0.11 a
DW (g)	shoot	0.151 ± 0.016 a	0.150 ± 0.023 a	0.146 ± 0.016 a	0.178 ± 0.019 a	0.156 ± 0.017 a
	4^th^ leaf	0.056 ± 0.005 a	0.048 ± 0.003 a	0.047 ± 0.004 a	0.055 ± 0.003 a	0.048 ± 0.005 a
	roots	0.045 ± 0.005 a	0.034 ± 0.004 a	0.037 ± 0.003 a	0.046 ± 0.004 a	0.039 ± 0.005 a
length (cm)	shoot	35.1 ± 1.7 a	32.0 ± 1.3 a	32.3 ± 1.2 a	35.0 ± 1.9 a	32.5 ± 1.6 a
	4^th^ leaf	26.9 ± 1.3 a	24.4 ± 0.8 a	24.4 ± 0.9 a	27.1 ± 1.6 a	25.1 ± 1.5 a
	roots	35.2 ± 2.1 a	32.3 ± 0.8 a	31.3 ± 0.9 a	32.9 ± 0.9 a	35.2 ± 1.3 a
node diameter (cm)	0.57 ± 0.02 a	0.56 ± 0.02 a	0.58 ± 0.03 a	0.60 ± 0.04 a	0.57 ± 0.02	
4^th^ leaf area (cm^2^)	34.6 ± 2.6 a	29.0 ± 1.1 a	28.7 ± 1.9 a	33.5 ± 2.5 a	30.0 ± 2.2 a	
LMA (g m^-2^)	185.4 ± 4.9 a	187.2 ± 8.1 a	175.8 ± 4.8 a	191.5 ± 7.3 a	188.8 ± 8.7 a	
**Accumulation of elements in plants (mg kg** ^**-1**^ **DW)**						
Ca	shoot	4,240 ± 320 b	2,630 ± 210 c	5,110 ± 120 a	4,140 ± 230 b	4,480 ± 210 b
	4^th^ leaf	2,940 ± 30 b	740 ± 90 c	3,480 ± 140 ab	3,630 ± 190 a	3,000 ± 180 b
	roots	4,810 ± 440 a	2,140 ± 60 b	5,130 ± 290 a	4,960 ± 90 a	4,960 ± 450 a
K	shoot	81,310 ± 2510 a	81,100 ± 2580 a	45,000 ± 1720 b	86,880 ± 2480 a	84,500 ± 1400 a
	4^th^ leaf	75,980 ± 930 a	78,500 ± 1820 a	38,980 ± 2230 b	80,400 ± 870 a	78,050 ± 1270 a
	roots	77,210 ± 1560 a	69,340 ± 2580 b	15,330 ± 600 c	79,430 ± 960 a	79,430 ± 2200 a
Mg	shoot	1,530 ± 40 a	1,700 ± 60 a	1,630 ± 30 a	1,010 ± 70 b	1,470 ± 20 a
	4^th^ leaf	1,400 ± 20 b	1,630 ± 60 a	1,460 ± 100 ab	810 ± 30 c	1,320 ± 30 b
	roots	1,440 ± 130 b	1,430 ± 70 b	1,780 ± 50 a	790 ± 10 c	1,790 ± 140 a
P	shoot	12,770 ± 320 a	12,980 ± 460 a	12,540 ± 570 a	12,370 ± 360 a	8,090 ± 360 b
	4^th^ leaf	9,300 ± 320 a	10,220 ± 300 a	9,310 ± 590 a	9,830 ± 220 a	5,340 ± 370 b
	roots	14,110 ± 420 a	13,980 ± 430 a	15,030 ± 460 a	14,570 ± 480 a	7,370 ± 690 b

Presented data are means ± SE (n = 8); Means followed by the same letter in a row are not significantly different from each other using the LSD test (P < 0.05).

### Chlorophyll *a* fluorescence

**Figure 1:**
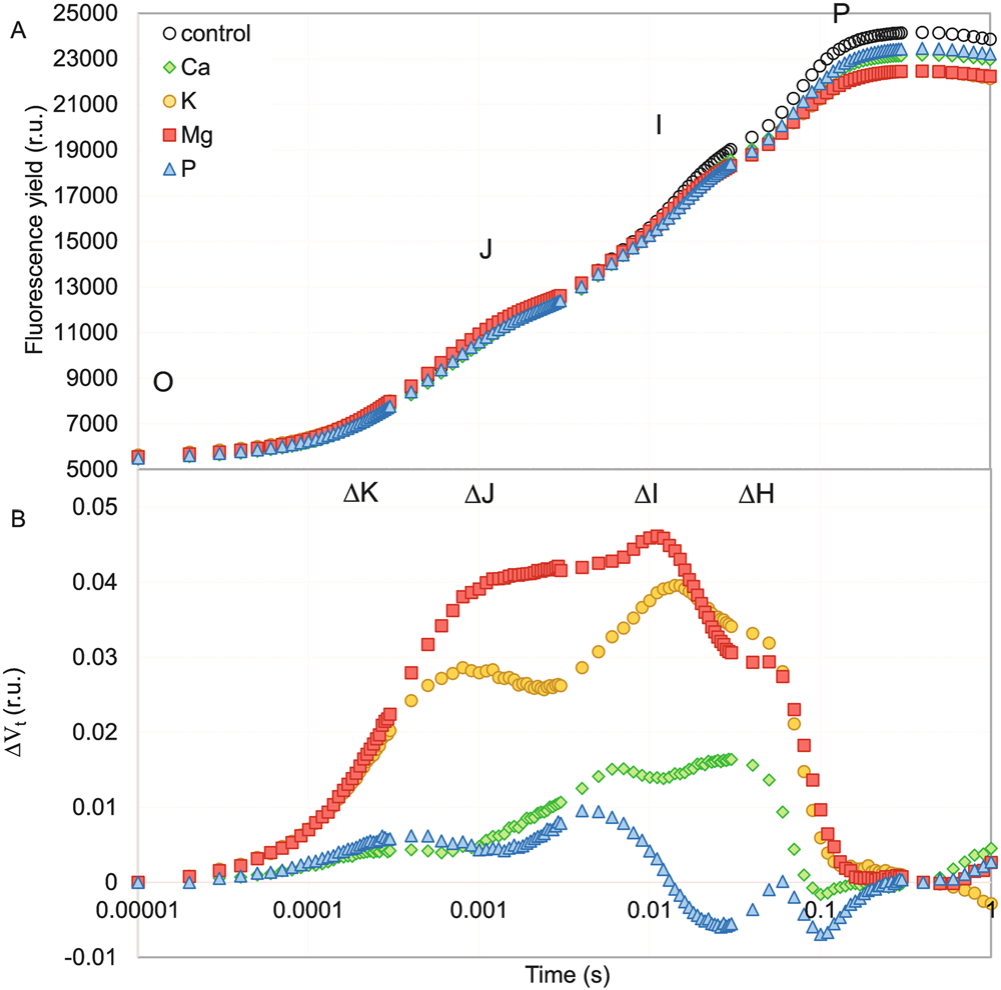
Chlorophyll *a* fluorescence induction curves of the *Zea mays* L. treated with different macronutrient deprivation (**A**) and the effect of these deprivation on the relative variable fluorescence (ΔV_t_ = ((F_t_ − F_0_)/F_v_) − V_t__control) (**B**). For ΔV_t_ analysis, the fluorescence of the control leaves was the reference and equaled 0. Values are means (n = 55).

**Figure 2:**
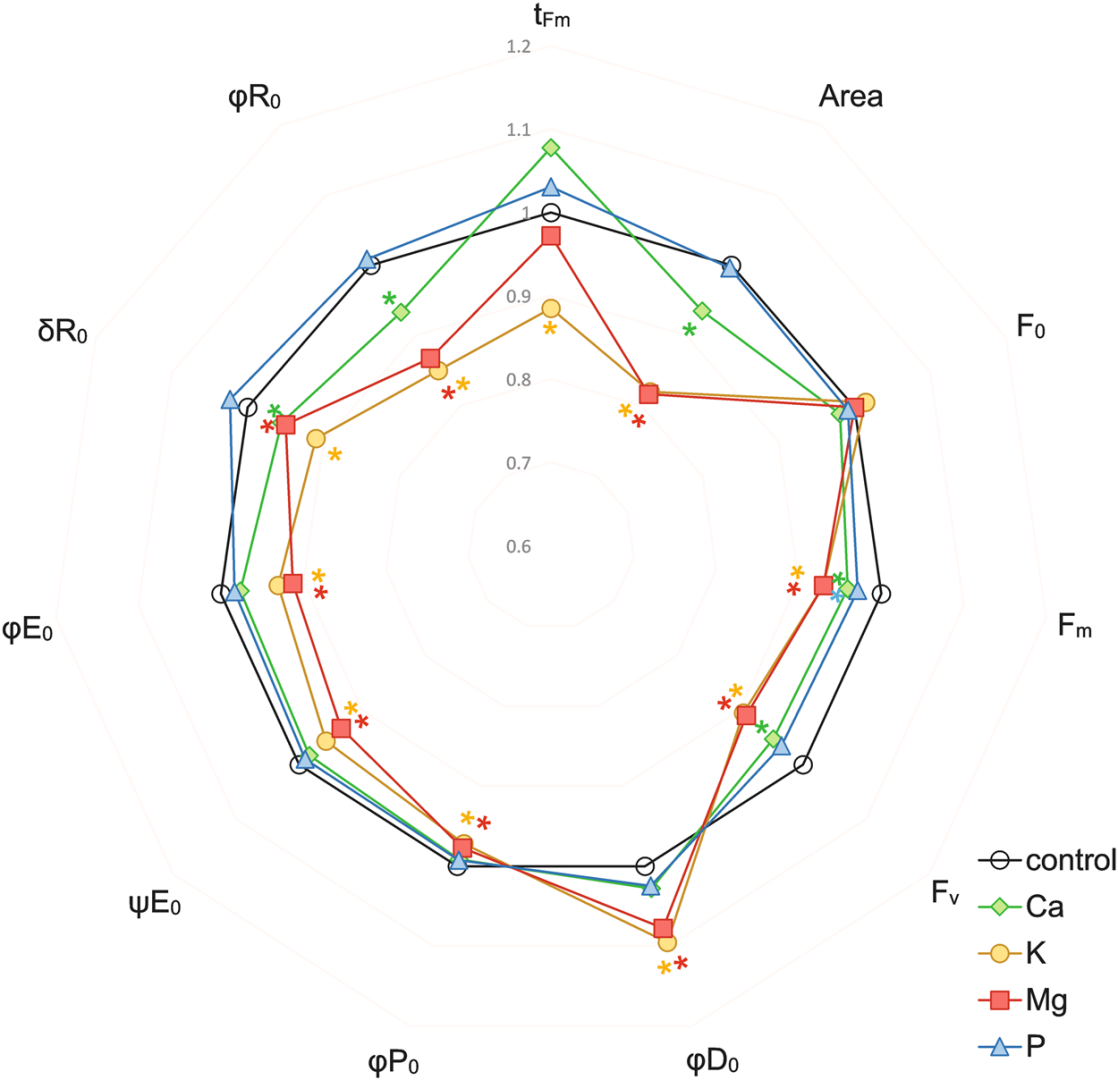
Relationships between parameters describing fluorescence and yield of photosystem II under macronutrient deprivation. Values are means (n = 55) after standardization. Asterisks describe significant differences compared with the control, using LSD test (P < 0.05). Parameters: t_Fm_ − time (in ms) to reach maximum fluorescence F_m_; Area − the area above the chlorophyll fluorescence curve between F_0_ and F_m_ (reflecting the size of the plastoquinone pool); F_0_ − minimum fluorescence; F_m_ − maximu_m_ fluorescence; F_v_ − maximum variable fluorescence; φP_0_ − maximum quantum yield of the primary PSII photochemistry; ΨE_0_ − probability (at time 0) that a trapped exciton moves an electron into the electron transport chain beyond Q_A_–; φE_0_ − quantum yield for electron transport from Q_A_– to plastoquinone; δR_0_ − probability with which an electron from the intersystem electron carriers will move to reduce the end acceptors at the PSI acceptor side; φR_0_ − quantum yield for the reduction of the end electron acceptors at the PSI acceptor side; φD_0_ − quantum yield (at t = 0) of energy dissipation.

Chlorophyll *a* fluorescence was presented both as typical OJIP curves and as a subtraction between the variable fluorescence curves of each deprivation variant and the control (∆Vt) (Fig. 1). One week of a macronutrient deprivation did not cause any evident deviations in the course of the fluorescence induction curves with the exception of a significant decrease in the maximum fluorescence (F_m_) (Figs 1A and 2). For ΔV_t_, there were four visible bands: ΔK (at ~300 μs), ΔJ (at ~2 ms), ΔI (at ~10 ms) and ΔH (at ~40 ms) (Fig. 1B). The Mg-deprived plants were characterized by the strongest negative changes, which were manifested as the presence of all of the abovementioned bands in the ΔV_t_ curve (Fig. 1B). A milder effect was observed in response to K starvation. Although all of the bands were also visible, the ΔJ was clearly smaller compared with the Mg-deprived plants. In the plants under Ca deprivation, only the ΔI and ΔH bands appeared and both were relatively small. On the other hand, the negative effect of a P deprivation was insignificant and the ΔV_t_ curve for the P-deprived maize was similar to the control (abscissa) (Fig. 1B).

The effect of macronutrient starvation on the selected parameters that describe the yield of the photosynthetic apparatus is presented in Fig. 2. The time that was required to reach the maximum fluorescence (t_Fm_) decreased significantly only in plants under K deprivation, whereas the total pool of plastoquinone (Area) was significantly decreased for the K-, Mg- and Ca-deprived plants compared with the control (Fig. 2). Moreover, the fact that there were no differences in F_0_ as well as a statistically significant decrease in F_m_ for all of the experimental variants compared to the control plants were in line with the observations of the fluorescence induction curves. Furthermore, the quantum yield and probability for electron transport from Q_A−_ to plastoquinone (φE_0_ and ψE_0_, respectively) and the maximum quantum efficiency of the PSII (φP_0_, for the control 0.77) were significantly decreased (for both Mg and K 0.75), whereas the quantum yield of energy dissipation (φD_0_) was increased only for the plants under K and Mg starvation. In the case of quantum yield and probability for the reduction of the end electron acceptors at the PSI acceptor side (φR_0_ and δR_0_, respectively), besides the K and Mg deprivation, a Ca deprivation also caused a considerable decrease in the parameters (Fig. 2), which additionally confirmed the observations of the ΔI and ΔH bands for this stress (Fig. 1B).

**Figure 3:**
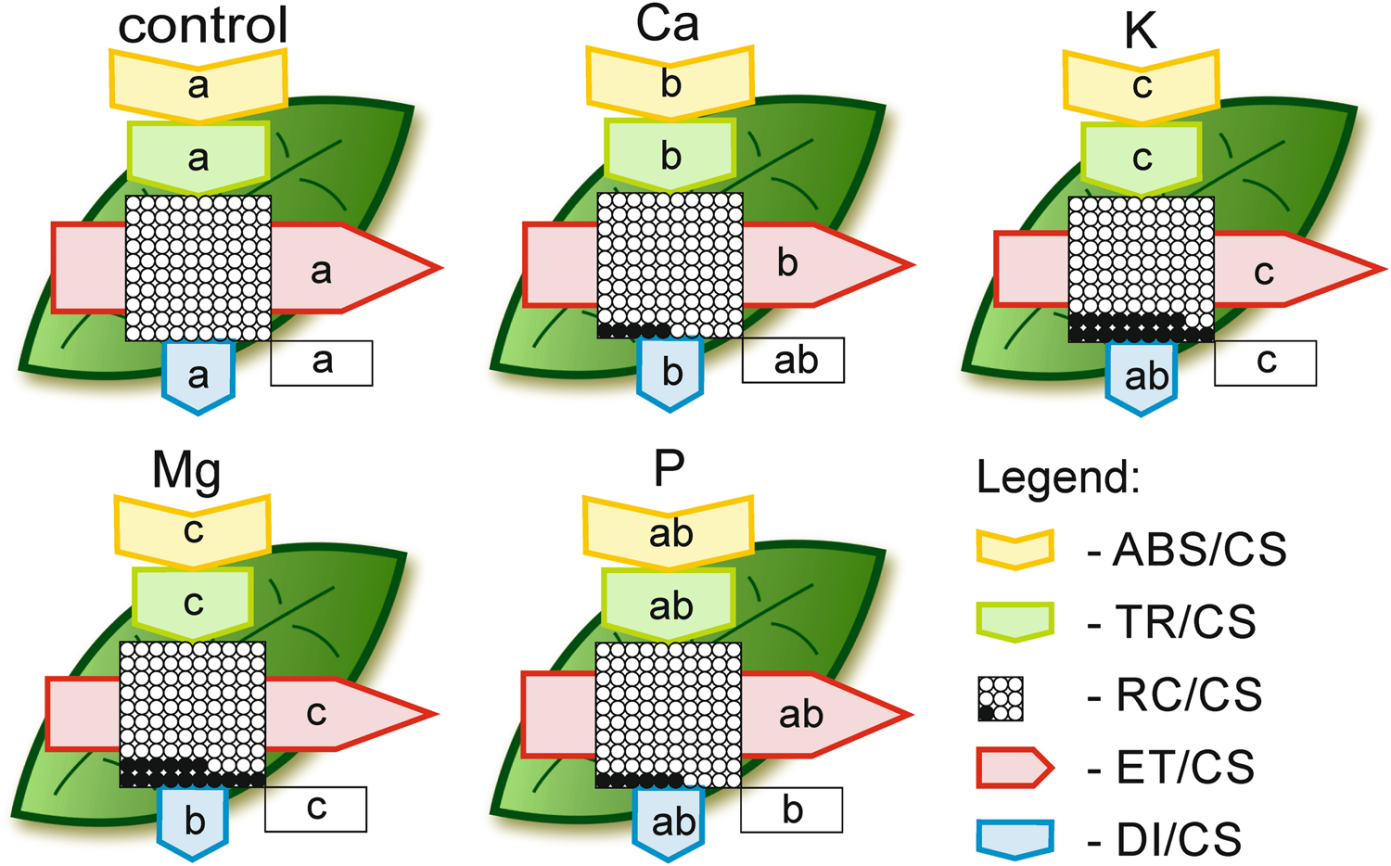
Leaf model showing the phenomenological energy fluxes per the excited cross sections (CS) of the leaves of the *Zea mays* L. under different macronutrient deprivation. Each relative value of the measured parameters is the mean (n = 55) and the width of each arrow corresponds to the intensity of the flux. Yellow arrow − ABS/CS, absorption flux per CS approximated; green arrow − TR/CS, trapped energy flux per CS; red arrow − ET/CS, electron transport flux per CS; blue arrow − DI/CS, dissipated energy flux per CS; circles inscribed in squares − RC/CS, % of active/inactive reaction centres. White circles inscribed in squares represent reduced Q_A_ reaction centres (active), black circles represent non-reducing Q_A_ reaction centres (inactive), 100% of the active reaction centres responded with the highest mean value observed in the control. Means followed by the same letter for each parameter are not significantly different from each other using the LSD test (P < 0.05). Letters are inscribed into arrows, except for RC/CS where they are placed in a box in the lower right corner of the square with circles.

The phenomenological models of energy fluxes through the cross sections (CS) of the leaves of *Zea mays* L. under different macronutrient deprivations are presented in Fig. 3. An Mg or K deprivation greatly diminished the energy fluxes in the plants compared to the other elements. Moreover, Ca starvation resulted in a significant decrease in the energy absorption by a cross section of the leaves (ABS/CS), energy trapping (TR/CS), the electron transport flux (ET/CS) and energy dissipation (DI/CS) compared with the control, but the decrease in all abovementioned parameters was significantly lower than the ones that were observed under K and Mg starvation. Furthermore, the Ca-deprived plants did not differ in the percentage of active reaction centres (RC) compared to the control plants. On the other hand, in P-deprived plants values of ABS/CS, TR/CS, DI/CS and ET/CS did not differ considerably compared to the control plants, except for the percentage of active reaction centres, which was significantly lower (Fig. 3).

### Photosynthetic rate and transpiration

**Figure 4:**
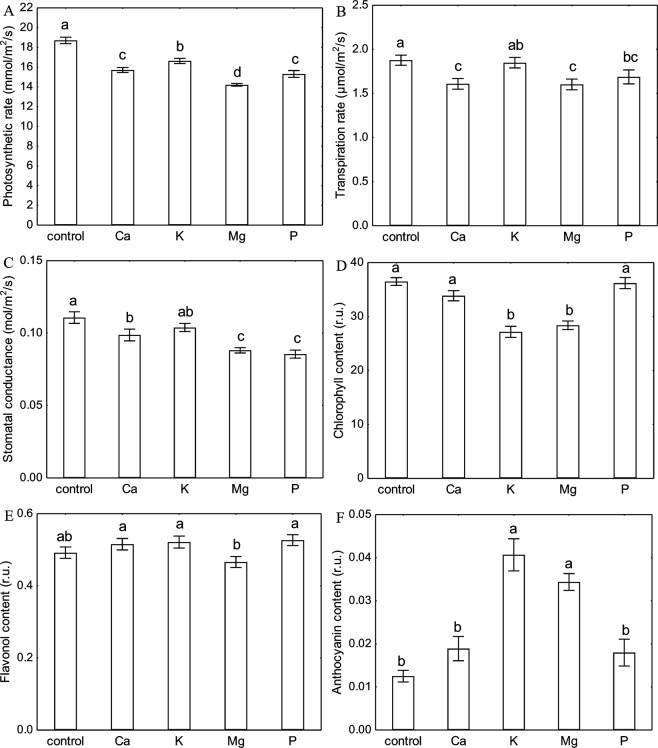
Changes in photosynthetic (**A**) and transpiration (**B**) rate, stomatal conductance (**C**) and chlorophyll (**D**), flavonol (**E**) and anthocyanin (**F**) content in leaves of *Zea mays* L. under different macronutrient deprivation. Presented values are means ± SE (n = 80, except pigments content, where n = 55). Means followed by the same letter in a single graph are not significantly different from each other using the LSD test (P < 0.05).

Each investigated macronutrient deprivation caused a significant decrease in the photosynthetic rate (Fig. 4A). The strongest inhibition of CO_2_ assimilation was exhibited by plants under Mg starvation (75% of control). The decrease in the photosynthetic rate of the Ca and P-deprived plants was similar and reached 84–81% of the control, whereas the plants under K starvation showed the lowest decrease in the photosynthetic rate (11%) compared with the control (Fig. 4A). The most significant inhibition of the transpiration rate by 15% was observed in the plants with a Ca and Mg deprivation (Fig. 4B). In contrast, the deprivation of K did not cause a significant decrease in either the transpiration rate or stomatal conductance (Fig. 4B,C). The Ca-deprived plants were characterized by a decrease in stomatal conductance by 11% compared with the control. The decrease in stomatal conductance in the Mg- and P-deprived plants was similar and reached 79 and 76% of the control, respectively (Fig. 4C).

### Pigment content

The only significant decrease in the chlorophyll content was measured in the leaves of the K- and Mg-deprived plants (74 and 78% of the control, respectively) (Fig. 4D). Interestingly, a significant increase of the anthocyanin content of 225% and 175% of the control was only observed for the K-and Mg-deprived plants, respectively (Fig. 4F). The flavonol content in the maize plants growing under conditions of a Ca, K and P deprivation was higher than the control, whereas in the Mg-deprived plants, a decrease in the flavonol content was observed; however, these differences were not statistically significant (Fig. 4E).

### Principal component analysis

**Figure 5:**
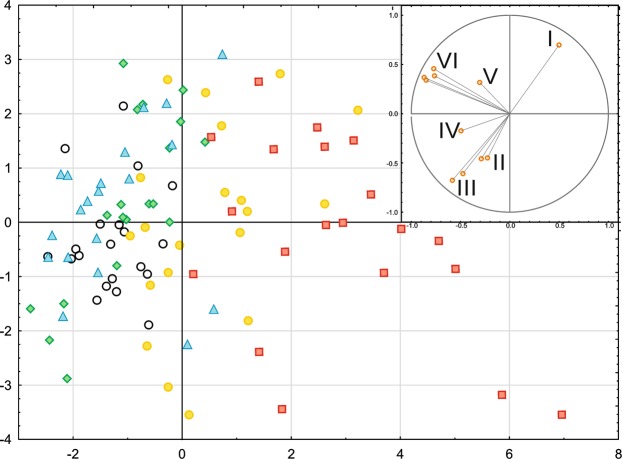
The Principal Component Analysis (PCA) presenting the relationships between selected photosynthetic parameters and pigment concentrations in maize under selected macronutrient deprivation. The variants are marked with points (white circle – control, green rhombus – Ca, yellow circle – K, red square – Mg, blue triangle – P,). The variables determining the distribution of the cases: I – Anthocyanin conent, II – Transpiration rate and stomatal conductance, III – Chlorophyll and flavonol content, IV – Photosynthetic rate, V - φD_0_ − quantum yield (at t = 0) of energy dissipation, VI − φP_0_, ψE_0_, φE_0_, δR_0_ and φR_0_.

A PCA analysis indicated a strong negative correlation between the anthocyanin content and the gas exchange parameters, especially the transpiration rate and stomatal conductance (Fig. 5). The parameters that describe the performance of the electron transport chain (φP_0_, ψE_0_, φE_0_, δR_0_, φR_0_, φD_0_) were clustered in a separate quarter, and therefore, they grouped the cases independent of the pigment content and gas exchange parameters. Based on the PCA analysis, we can conclude that in the case of one-week-long deprivations most affected photosynthetic apparatus was observed in Mg- and K-deprived plants. An Mg and K deprivation were closely associated with high anthocyanin content and a decrease in most photosynthetic efficiency parameters simultaneously. Conversely, the plants under P starvation showed the lowest amount of damage for all of the conditions that were tested compared with the control (Fig. 5). In addition, the differences between measured physiological parameters of macroelement deprivation-stressed plants are presented in Table 2.

**Table 2:**
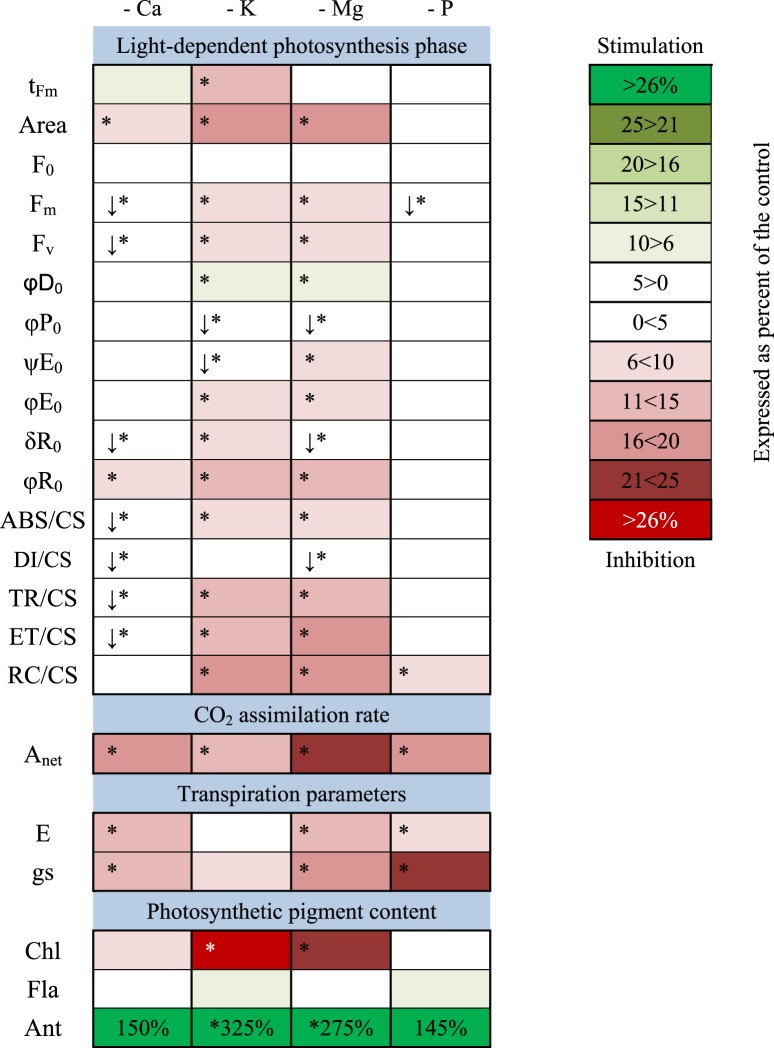
Changes in parameters describing photosynthetic apparatus performance of maize under deprivation of Ca, K, Mg or P.

Asterisks show the statistical significance for each parameter compared with the control (ANOVA, LSD test, P < 0.05). Arrows describe the direction of statistically significant change when it is in the range ± 5% of the control.

## Discussion

The problem of a nutrient deprivation has been well known for years and has also been relatively well examined; however, there is a lack of research that investigates the effect of starvation on both light and dark reactions of photosynthesis simultaneously. Moreover, the description of chlorophyll *a* fluorescence in many reports are often limited to only a few parameters, which do not reflect the possibilities of the method. Furthermore, in most cases of a nutrient deprivation, there is a dearth of data that characterize changes in the content of flavonols or anthocyanins, which are stress markers and can be easily determined today. Here, we present a wide spectrum of analyses that were focused on the processes connected with photosynthesis in maize under macronutrient starvation conditions.

One of the goals of this work was to find physiological markers of short-term macronutrient deprivation. For this reason plant seedlings were subjected to nutrient deprivation stress only for seven days, in contrast to experiments conducted by other authors^17–21,24,34–37^. We found that such short-term treatment caused in shoots and roots a significant decrease in concertation of a macronutrient removed from the solution (Table 1). However, no one of investigated growth parameters was lowered in nutrient-deprived plants (Table 1). The deficiency phenomenon is featured by the critical concentration of element, below which growth and production of plant are adversely affected. Strammer and Mallarino^38^ presented the relationship between corn relative grain yield and the P or K concentration in shoots of plants, which were grown in field conditions. They found, that the critical concentration range of P and K was from 4,800 to 5,500 and 18,800 to 25,300 mg kg^−1^, respectively. Thus, the results presented in the current study did not show the occurrence of deficiency of both macronutrients in tested plants (Table 1).

There are a few reports that have described the impact of Ca deprivation on photosynthesis in detail. Kalaji *et al*.^37^ found that Ca starvation caused the highest and most significant changes among seven tested variants of nutrient deprivation (14-day-long deficiencies of Ca, S, Mg, K, N, P and Fe) in the ΔV_t_ curve and most of OJIP test parameters for maize, whereas for tomato, the changes that were observed were not so extreme. A decrease in the chlorophyll content (in coffee tree, tomato and rice) and the maximum quantum efficiency of PSII (φP_0_) (in coffee tree and tomato) under Ca starvation stress have also been documented by other authors^39–41^. We found that seven days of Ca deprivation primarily affected the electron transport to PSI (I-P phase) (Fig. 1B), which was also confirmed by a significant decrease of δR_0_ and φR_0_ (Fig. 2). The obtained results may be connected with the Ca^2+^-dependent regulation of the biosynthesis of NADP^+^ ^29^. We also observed a significant decrease in the parameters of the energy fluxes such as energy absorbance (ABS/CS), trapped energy (TR/CS), electron transport flux (ET/CS) and dissipated energy (DI/CS) per a cross section of maize leaf (except RC/CS) (Fig. 3) under Ca deprivation for the first time. Liang *et al*.^42^ showed that cucumber leaves that had been sprayed with calcium chloride were characterized by a significantly higher photosynthetic rate than plants that were grown without calcium. Our results confirmed that Ca-deprived plants had a significantly lower photosynthetic rate compared to plants that were grown in a full nutrient medium (Fig. 4B). However, the results of the measurements did not indicate a negative effect of a deprivation of Ca on the chlorophyll content (Fig. 4D), which corresponds with the results that were obtained by Cho *et al*. for rice^41^. On the other hand, Kalaji *et al*.^43^ observed a decrease in the chlorophyll content in maize under Ca deprivation by 55% of the control, which may have been caused by a longer (14 days) period of starvation. Although there is a lack of published data that describe the impact of a Ca deprivation on the content of flavonols and anthocyanins in leaves, the results of present study suggest an insignificant increase of the content of both pigments under short-term Ca starvation (Fig. 4E,F).

Kalaji *et al*.^37^ documented that two weeks of K starvation resulted in the presence of a relatively low positive ΔK band with a long shoulder to the ΔH band in maize, while in tomato, the ΔK band was the highest for all of the seven tested variants of nutrient deficiencies and the ΔI was the final observed positive band on the ΔV_t_ curve. In addition, changes in the OJIP test parameters confirmed that relationships were observed for the ΔV_t_ curves for both species^37^. Our results showed that one week of K starvation resulted in the appearance of a relatively high positive ΔI band with a shoulder to the ΔH band. Moreover, a positive ΔJ band was observed, which may suggest disturbances in the performance of both photosystems (Fig. 1B). We observed a significant decrease in all of the investigated OJIP test parameters except for a significant increase of φD_0_ and no differences in F_0_ compared with the control (Fig. 2). A significant decrease in ABS/CS, TR/CS, ET/CS and in the percentage of active reaction centres per CS (Fig. 3) are presented for the first time and they confirm the negative effect of a K deprivation on the energy fluxes in PSII. These observations suggest a key role of K in the correct functioning of the electron transport chain. Many reports have shown the inhibiting effect of a K deprivation on the gas exchange parameters, in long-term K deprivation experiments, a decrease has invariably been observed for both the photosynthetic rate and stomatal conductance^17–21,24,35,36^. In contrast, our results indicate that a short-term K starvation significantly decreased only the photosynthetic rate (Fig. 4A), while the decreases in the transpiration rate and stomatal conductance were insignificant compared with the control (Fig. 4B,C). Many authors have also observed that a K deprivation decreased the chlorophyll content^21,34,37^, which is also in agreement with our results (Fig. 4D). Hodges and Nozzolillo^44^ examined several plant species (cabbage, cauliflower, radish and canola) for changes in the anthocyanin content under some types of nutrient deprivation stress and they found no significant differences in the anthocyanin content for any of the investigated species under K starvation. In contrast, the results of the current study indicate a strong effect of a K deprivation on the increase of anthocyanins in the leaves of maize, which suggests that this may be a species-specific reaction to stress (Fig. 4F). While there is a lack of published data that show the effect of K starvation on the flavonol content in the leaves of any plant species, our results indicate an insignificant increase of the content of these pigments compared with the control (Fig. 4E).

Most of the known functions of Mg in photosynthesis are that it is the central atom of the chlorophyll molecule^45^, hence plants have strong reactions to a deprivation of this macronutrient. A deprivation of Mg usually causes strong response in the chlorophyll *a* fluorescence, which is visible as the presence of positive bands on the ΔV_t_ curve^3,37^. Kalaji *et al*.^37^ observed high positive ΔK, ΔJ, ΔI and ΔH bands for maize and relatively low positive ΔK and ΔI bands for tomato under 14 days of Mg deprivation, while Samborska *et al*.^3^ found the presence of only a positive ΔJ band after seven days of a Mg deprivation in radish. Our results showed that seven days of Mg starvation caused disturbances in both O-J (ΔJ band with a shoulder to the ΔK band) and I-P (the highest ΔI band with a shoulder to the visible ΔH band) phases of the relative variable chlorophyll *a* fluorescence induction curves (Fig. 1B). Moreover, Kalaji *et al*.^37^ observed a decrease in most of the parameters that describe the OJIP test (e.g. φP_0_, ψE_0_, φE_0_, δR_0_ and φR_0_) and a characteristic increase of φD_0_ that was caused by Mg starvation in maize, which is also confirmed by our results (Fig. 2). The negative effect of a deprivation of Mg on the energy fluxes per an excited cross section in PSII was also presented on the leaf models (Fig. 3). So far, to the best of our knowledge, no plant research studies have been published, which present influence of Mg deficiency on these parameters. The Mg starvation significantly affected photosynthesis, the transpiration rate and stomatal conductance, which has been confirmed by many authors^3,46–48^ and was also observed in our research (Fig. 4A–C). Furthermore, Samborska *et al*.^3^ found a significant decrease in the chlorophyll *a* content but no significant changes in the flavonol content in the leaves of radish under an Mg deprivation, which is also confirmed by our results for maize (Fig. 4D,E). In this study, we showed, for the first time, that a deprivation of Mg enhanced the anthocyanin content, which suggest that this parameter may be a species-specific stress marker in maize for this type of stress (Fig. 4F).

Kalaji *et al*.^37^ found that a 14-day-long P starvation of maize plants caused relatively minor changes in the ΔV_t_ curve course and in the OJIP test parameters (the lowest and most insignificant changes among the seven tested variants of nutrient deficiencies). Our work confirmed these results, and moreover, the ΔV_t_ curve for the P-deprived maize plants was characterized by a similar shape to observed by Kalaji *et al*. (2014) (Fig. 1B), and no significant changes were observed in the OJIP test parameters except for a decrease of F_m_ (Fig. 2). Furthermore, Carstensen *et al*.^49^ showed that a P deprivation primarily affects the electron transport to photosystem I (PSI), which can be detected based on the depletion of the I-step from the fast OJIP region of the chlorophyll *a* fluorescence transient. A similar observation of a fading I-step in plants suffering from a P deprivation was described by Frydenvang *et al*.^50^. The results presented in the current study confirm that the only significant observed band for the P-deprived plants was the positive ΔI band (Fig. 1B), which suggest that there were disturbances in the I-P phase of the OJIP transient. This result may be correlated with a restricted PQH_2_ oxidation due to the proton backlog that is caused by lumen acidification as was proposed by Carstensen *et al*.^49^. Many studies have reported a decrease in the photosynthetic and transpiration rates under P deprivation stress^51–53^. The presented data also confirm a significant decrease in these parameters (Fig. 4A–C) that was caused by P starvation. Xu *et al*.^54^ found that eight days of a P deprivation did not result in a decrease of the chlorophyll content in the leaves of rice. Similar results were obtained for soybean^6^ and maize^43^. Our results also showed that one week of P deprivation stress did not result in a decrease in the chlorophyll content (Fig. 4D). In contrast, a significant increase in the anthocyanin content in maize leaves after 25 days of P starvation was observed by Sun *et al*.^55^. Moreover, Hodges and Nozzolillo^44^ found an increase of the anthocyanin content in cabbage, cauliflower and canola under a deprivation of P. The presented data confirm an increase of the anthocyanin content by 44% of the control in the leaves of P-deprived maize (Fig. 4F); however, this increase was not statistically significant. An enhancement in the flavonol content with an increasing P deprivation in a medium for *Arabidopsis thaliana* and *Lycopersicon esculentum* was observed by Stewart *et al*.^56^. Our results also indicated that the increase in the flavonol content was caused by P starvation, but it was not statistically significant (Fig. 4E).

In the research presented here, we examined of selected macronutrient deprivation (Ca, K, Mg, P), on the following parameters: growth, the light-dependent and light-independent phases of photosynthesis, transpiration, stomatal conductance and pigment content in maize simultaneously for the first time. It should be emphasized that one week starvation did not caused deficiency of any macronutrient and growth inhibition. We found that the short-term removal of Mg, K and Ca had the most negative effect on the light-dependent phase of photosynthesis. On the other hand, the gas exchange was mostly inhibited by a deprivation of Mg, P and Ca. By contrast, K starvation did not result in any significant changes in transpiration and stomatal conductance compared with the control and that the decrease in the photosynthetic rate was the smallest compared to all of the investigated macronutrients. Interestingly, the K-deprived plants were characterized by the highest anthocyanin content, while the second and last significant increases in these pigments were observed for Mg deprivation. An inverse relationship was observed for changes in the chlorophyll content. We show for the first time how short term macronutrient deprivation affect the energy fluxes per a cross section and the content of flavonols and anthocyanins in the leaves of maize. As a result, this work substantially expands our knowledge of the effect of macronutrient deprivation on both the light-dependent and light-independent phases of photosynthesis and provides new data on the relationships between abovementioned processes and changes in the content of anthocyanins and flavonols. Based on the presented data, we can conclude, that the set of measurements that were used in the current study made it possible to determine the specific plant responses to each short-time macronutrient deprivation stress. We also documented that alterations in photosynthesis, transpiration or pigment content, as a response to macronutrient deprivations, are faster than growth inhibition or nutrient deficiency, suggesting that these alterations may be considered as an early-acting stress sensors.

## Materials and Methods

### Plant material

Caryopses of *Zea mays* L. cv. ‘Lokata’ were soaked in tap water for 2 h and sown in plastic trays lined with moistened cellulose sheets and germinated in an incubator (MIR-533, SANYO, Japan) at a temperature of 27 ± 1 °C in darkness. After three days, seedlings with 3.5 ± 0.5 cm long coleoptiles and a well-developed primary root were selected and transferred into the hydroponic culture.

### Hydroponic culture

The seedlings were cultivated using a standard Hoagland medium^57^ for the first two weeks of development with nine plants per container filed with 2,850 ml (315 ml/seedling) of the nutrient solution. The medium was changed twice a week and constantly aerated. The composition of the macronutrients is presented in Table 3. The micronutrients remained unchanged (25 µM B, 2 µM Mn, 2 µM Zn, 0.5 µM Cu, 0.5 µM Mo and 50 µM Fe). The initial medium pH was adjusted to 6.0 ± 0.1 with 0.1 M HCl or 0.1 M NaOH. The average temperature in the greenhouse for day/night was 21/18 °C, respectively, the relative humidity was 30–40%, the photoperiod for the day/night cycle was 16/8 h and the minimum photosynthetically active radiation, which was provided by an HPS lamps was 300 ± 20 µmol m^−2^ s^−1^. After 14 days of growth, the seedlings were subjected to nutrient deprivation stress (Table 3.). At the 7^th^ day of the stress treatment, the measurements were performed. To compensate for the lack of S in the ‘- Mg’ medium and the lower concentration of N in the ‘- Ca’ and ‘- K’ medium, the appropriate amount of Na_2_SO_4_ or NaNO_3_ was introduced into the nutrient solution (Table 3).

**Table 3:** The composition of the various culture media used in the experiment.

	Type of growth medium (mM).				
	Full	-Ca	-K	-Mg	-P
Ca(NO_3_)_2_ ∙ 4H_2_O	4	—	4	4	4
KNO_3_	6	6	—	6	6
MgSO_4_ ∙ 7H_2_O	2	2	2	—	2
NH_4_H_2_PO_4_	1	1	1	1	—
NaNO_3_	—	8	6	—	—
Na_2_SO_4_	—	—	—	2	—

### Plant growth parameters and accumulation of elements

Plant growth parameter measurements were performed at the end of the experiment. Length of shoots, 4^th^ leaves and roots was measured using ruler. The node diameter was measured using callipers. After elongation measurements the fresh weight of shoots, 4^th^ leaves and roots was measured using analytical balance (SA 210, Scientech Inc., USA). The area of each 4^th^ leaf was determined using scanner (GT-1500, Epson, Japan) and ImageJ software. The plant samples were packed into paper envelopes and dried in 70 °C for three days in a drier (WGLL-65BE, ChemLand, Poland), than the samples were weighed using the same analytical balance. The dry plant material for each sample was ground in a mortar, mixed, and acid digested in a microwave-assisted wet digestion system (ETHOS1, Milestone, Italy) according to the procedure provided by the manufacturer (concentrated HNO_3_ and H_2_O_2_, 4:1, v/v). The concentration of metals was analysed in the digests using flame atomic absorption spectrophotometry (iCE 3500 FAAS; Thermo Scientific). Reference plant material (Oriental Basma Tobacco Leaves [INCT-OBTL-5]; Institute of Nuclear Chemistry and Technology) was used for the quality assurance of the analytical data.

### Gas exchange, chlorophyll a fluorescence and pigment content measurements

The measurements were taken in the greenhouse under the same temperature and humidity conditions as during the growth of plants. All of the measurements were performed on the 4^th^ fully developed leaf, which was counted from the base of the stem for eight plants from each treatment. We found that the 4^th^ leaf was optimal due to its intense growth in the nutrient deprivation phase. Gas exchange was measured with an infrared gas analyser (LCpro^+^, ADC BioScientific Ltd, UK) with a light intensity of 1,000 µmol m^−2^ s^−1^ in the measurement chamber. Five measurements were performed for each leaf after a three-minute acclimatization in the chamber. The chlorophyll *a* fluorescence was measured with a fluorimeter (PocketPEA, Hansatech Instruments Ltd., England). Three measurements (at the base, middle and subapical part of leaf) were performed after a 30-minute acclimatisation in the dark using special leaf clips (Hansatech Instruments Ltd., England) for each measured leaf. Chlorophyll *a* fluorescence was recorded after illumination using a red actinic light (635 nm, 3,500 μmol m^−2^ s^−1^) for one second. The content of chlorophyll, anthocyanins and flavonols were measured with a Dualex Scientific + (Force-A, France) sensor. Five measurements (at the base, middle and subapical part of a leaf) were performed for each leaf. The experiment was repeated twice.

### Statistical analyses

The results are shown as the means ± SE. The statistical significance of the differences was determined using a one-way ANOVA and the post-hoc LSD test (P < 0.05). PCA was used to identify the dominant groups of the factors that determine a macronutrient deprivation. The software that was used for the statistical analyses was Statistica v. 13.1 (Dell Inc., USA). The pipeline models of energy fluxes through the leaf CSs were created using CorelDRAW X6 (Corel Corp., Canada).

## Supplementary information

Supplementary table

## Data Availability

The datasets generated during and/or analysed during the current study are available from the corresponding author on reasonable request.

## References

[1] Mengel, K., Kirkby, E.A., Kosegarten, H. & Appel, T. Principles of plant nutrition. fifth ed. Kluwer Academic Publishers Dordrecht The Netherlands (2001).

[2] Hawkesford, H. *et al*. Functions of macronutrients. In: Marschner P. (ed.), Marschner’s mineral nutrition of higher plants 3rd ed. UK, Elsevier Ltd., 135–189 (2012).

[3] Samborska IA (2018). Structural and functional disorder in the photosynthetic apparatus of radish plants under magnesium deprivation. Funct. Plant Biol..

[4] Blankenship, R. E. The basic principles of photosynthetic energy storage. In: Molecular mechanisms of photosynthesis; first edn. Wiley., pp 1–9 (2014).

[5] Engels, C., Kirkby, E., White, P. Mineral nutrition, yield and source-sink relationships. In: Marschner P. (ed.), Marschner’s mineral nutrition of higher plants 3rd ed. UK, Elsevier Ltd., 85–131 (2012).

[6] Singh SK, Reddy VR, Fleisher DH, Timlin DJ (2017). Relationship between photosynthetic pigments and chlorophyll fluorescence in soybean under varying phosphorus nutrition at ambient and elevated CO_2_. Photosynthetica.

[7] Amtmann A, Armengaud P (2009). Effects of N, P, K and S on metabolism: new knowledge gained from multi-level analysis. Curr. Opin. Plant Biol..

[8] Maathuis F (2009). Physiological functions of mineral macronutrients. Curr. Opin. Plant Biol..

[9] Yan N (2015). Changes in plant growth and photosynthetic performance of *Zizania latifolia* exposed to different phosphorus concentrations under hydroponic condition. Photosynthetica.

[10] Bose J, Babourina O, Rengel Z (2011). Role of magnesium in alleviation of aluminium toxicity in plants. J. Exp. Bot..

[11] Verbruggen N, Hermans C (2013). Physiological and molecular responses to magnesium nutritional imbalance in plants. Plant Soil..

[12] Tatagiba SD, DaMatta FM, Rodrigues FA (2016). Magnesium decreases leaf scald symptoms on rice leaves and preserves their photosynthetic performance. Plant Physiol. Bioch.

[13] Longstreth DJ, Nobel PS (1980). Nutrient influences on leaf photosynthesis. Effects of nitrogen, phosphorus and potassium for *Gossypium hirsutum* L. Plant Physiol..

[14] Huber SC (1984). Biochemical basis for effects of K-deprivation on assimilate export rate and accumulation of soluble sugars in soybean leaves. Plant Physiol..

[15] Bednarz CW, Oosterhuis DM (1999). Physiological changes associated with potassium deprivation in cotton. J. Plant Nutr..

[16] Erel R (2015). Modification of non-stomatal limitation and photoprotection due to K and Na nutrition of olive trees. J. Plant Physiol..

[17] Weng X-Y, Zheng C-J, Xu H-X, Sun J-Y (2007). Characteristics of photosynthesis and functions of the water–water cycle in rice (*Oryza sativa*) leaves in response to potassium deprivation. Physiol. Plant..

[18] Battie-Laclau P (2014). Photosynthetic and anatomical responses of *Eucalyptus grandis* leaves to potassium and sodium supply in a field experiment. Plant Cell Environ..

[19] Jákli B, Tavakola E, Tränknera M, Senbayrama M, Dittert K (2017). Quantitative limitations to photosynthesis in K deficient sunflower and their implications on water-use efficiency. J. Plant Physiol..

[20] Singh SK, Reddy VR (2018). Co-regulation of photosynthetic processes under potassium deprivation across CO_2_ levels in soybean: mechanisms of limitations and adaptations. Photosynth. Res..

[21] Zhao D, Oosterhuis DM, Bednarz CW (2001). Influence of potassium deprivation on photosynthesis, chlorophyll content and chloroplast ultrastructure of cotton plants. Photosynthetica.

[22] Cakmak I (2005). The role of potassium in alleviating detrimental effects of abiotic stresses in plants. J. Plant Nutr. Soil Sci..

[23] Blatt MR (1990). Potassium channel currents in intact stomatal guard cells: rapid enhancement by abscisic acid. Planta.

[24] Egilla JN, Davies FT, Boutton TW (2005). Drought stress influences leaf water content, photosynthesis and water-use efficiency of *Hibiscus rosa-sinensis* at three potassium concentrations. Photosynthetica.

[25] Tomemori H, Hamamura K, Tanabe K (2002). Interactive effects of sodium and potassium on the growth and photosynthesis of spinach and komatsuna. Plant Prod. Sci..

[26] Jin SH (2011). Effects of potassium supply on limitations of photosynthesis by mesophyll diffusion conductance in *Carya cathayensis*. Tree Physiol..

[27] Wang M, Zheng Q, Shen Q, Gu S (2013). The critical role of potassium in plant stress response. Int. J. Mol. Sci..

[28] White PJ, Broadley MR (2003). Calcium in Plants. Ann. Bot..

[29] Hochmal AK, Schulze S, Trompelt K, Hippler M (2015). Calcium-dependent regulation of photosynthesis. Biochim. Biophys. Acta.

[30] Dolatabadian A (2013). The role of calcium in improving photosynthesis and related physiological and biochemical attributes of spring wheat subjected to simulated acid rain. Physiol. Mol. Biol. Plants.

[31] Zhang G (2014). Exogenous calcium alleviates low night temperature stress on the photosynthetic apparatus of tomato leaves. PLoS ONE.

[32] Tan W, Meng QW, Brestic M, Olsovska K, Yang X (2011). Photosynthesis is improved by exogenous calcium in heat stressed tobacco plants. J. Plant Physiol..

[33] Liu YF, Zhang GX, Qi MF, Li TL (2015). Effects of Calcium on Photosynthesis, Antioxidant System and Chloroplast Ultrastructure in Tomato Leaves Under Low Night Temperature Stress. J. Plant Growth Regul..

[34] Reddy KR, Zhao D (2005). Interactive effects of elevated CO_2_ and potassium deprivation on photosynthesis, growth and biomass partitioning of cotton. Field Crops Res..

[35] Degl’Innocenti E, Hafsi C, Guidi L, Navari-Izzo F (2009). The effect of salinity on photosynthetic activity in potassium-deficient barley species. J. Plant Physiol..

[36] Kanai S (2011). Potassium deprivation affects water status and photosynthetic rate of the vegetative sink in green house tomato prior to its effects on source activity. Plant Sci..

[37] Kalaji HM (2014). Identification of nutrient deprivation in maize and tomato plants by *in vivo* chlorophyll *a* fluorescence measurements. Plant Physiol. Bioch..

[38] Strammer AJ, Mallarino AP (2018). Plant tissue analysis to assess phosphorus and potassium nutritional status of corn and soybean. Soil Sci. Soc. Am. J..

[39] Ramalho JC, Rebelo MC, Emilia Santos M, Luisa Antunes M, Antonieta Nunes M (1995). Effects of calcium deprivation on *Coffea Arabica*. Nutrient changes and correlations of calcium levels with some photosynthetic parameters. Plant Soil.

[40] Schmitz-Eiberger M, Haefs R, Noga G (2002). Calcium deprivation: influence in the antioxidative defense system in tomato plants. J. Plant Physiol..

[41] Cho S-C, Chao YY, Kao CH (2012). Calcium deprivation increases Cd toxicity and Ca is required for heat-shock induced Cd tolerance in rice seedlings. J. Plant Physiol..

[42] Liang W, Wang M, Ai X (2009). The role of calcium in regulating photosynthesis and related physiological indexes of cucumber seedlings under low light intensity and supoptimal temperature stress. Sci. Hort..

[43] Kalaji HM (2017). A comparison between different chlorophyll content meters under nutrient deprivation conditions. J. Plant Nutr..

[44] Hodges DM, Nozzolillo C (1996). Anthocyanin and anthocyanoplast content of cruciferous seedlings subjected to mineral nutrient deficiencies. J. Plant Physiol..

[45] Shaul O (2002). Magnesium transport and function in plants: the tip of the iceberg. BioMetals.

[46] Laing W (2000). Physiological impacts of Mg deprivation in *Pinus radiata*: growth and photosynthesis. New Phytol..

[47] Lasa B (2000). Effects of low and high levels of magnesium on the response of sunflower plants grown with ammonium and nitrate. Plant Soil.

[48] Hao X, Papadopoulos AP (2003). Effects of calcium and magnesium on growth, fruit yield and quality in a fall greenhouse tomato crop grown on rockwool. Can. J. Plant Sci..

[49] Carstensen A (2018). The Impacts of Phosphorus Deprivation on the Photosynthetic Electron Transport Chain. Plant Physiol..

[50] Frydenvang J (2015). Sensitive detection of phosphorus deprivation in plants using chlorophyll *a* fluorescence. Plant Physiol..

[51] Wissuwa M, Gamat G, Ismail AM (2005). Is root growth under phosphorus deprivation affected by source or sink limitations?. J. Exp. Bot..

[52] Xing D, Wu Y (2014). Effect of phosphorus deprivation on photosynthetic inorganic carbon assimilation of three climber plant species. Bot. Stud..

[53] Veronica N (2017). Influence of low phosphorus concentration on leaf photosynthetic characteristics and antioxidant response of rice genotypes. Photosynthetica.

[54] Xu HX, Weng XY, Yang Y (2007). Effect of Phosphorus Deprivation on the Photosynthetic Characteristics of Rice Plants. Russ. J. Plant Physiol..

[55] Sun Y (2016). Comparative transcript profiling of maize inbreds in response to long-term phosphorus deprivation stress. Plant Physiol. Bioch..

[56] Stewart AJ (2001). The effect of nitrogen and phosphorus deprivation on flavonol accumulation in plant tissues. Plant Cell Environ..

[57] Bloom, A. J. Mineral nutrition. In: Plant physiology and development, Sixth edition, Taiz L., Zeiger E., Møller I. M. and Murphy A. (eds), Sinauer Associates, Inc., Sunderland, USA, 119–142 (2015).

