# Supplementary material for: Influence of short-term macronutrient deprivation in maize on photosynthetic characteristics, transpiration and pigment content
Source: Sci Rep. 2019 Oct 2;9:14181. doi: 10.1038/s41598-019-50579-1 (PMC6775257; doi:10.1038/s41598-019-50579-1)
Supplement: Supplementary file 1 — Supplementary table [file 41598_2019_50579_MOESM1_ESM.docx]

| **Table S1.** Abbreviations | |
| --- | --- |
| General terminology | |
| PSII | - photosystem II |
| Q_A_ | - primary quinone electron acceptors of PSII |
| OJIP | - transient chlorophyll a fluorescence rise induced during a dark-to-strong light transition, where O is equivalent to F_0_ and P is for peak equivalent to F_m_ |
| CS | - excited cross section of leaf |
| RC | - reaction centre of PSII |
| Fluorescence parameters | |
| F_0_ | - minimal fluorescence, when all PS II RCs are open (at t = 0) |
| F_m_ | - maximal fluorescence, when all PS II RCs are closed |
| F_v_ | - maximal variable fluorescence |
| F_t_ | - fluorescence at time t |
| V_t_ | - relative variable fluorescence at time t |
| ΔV_t_ | = Vt - Vt_control |
| t_Fm_ | - time (s) to reach maximal fluorescence F_m_ |
| Area | - total complementary area between fluorescence induction curve and F = F_m_ (total plastoquinone pool) |
| Yields or flux ratios | |
| φP_0_ | - maximum quantum yield of primary photochemistry (at t = 0) |
| ΨE_0_ | - probability (at t = 0) that a trapped exciton moves an electron into the electron transport chain beyond Q_A_ |
| φE_0_ | - quantum yield of electron transport (at t = 0) |
| δR_0_ | - probability with which an electron from the intersystem electron carriers will move to reduce the end acceptors at the PSI acceptor side |
| φR_0_ | - quantum yield for reduction of end electron acceptors at the PSI acceptor side |
| φD_0_ | - quantum yield (at t = 0) of energy dissipation |
| Phenomenological energy fluxes (per excited cross section) | |
| ABS/CS | - absorption flux per CS |
| TR/CS | - trapped energy flux per CS |
| ET/CS | - electron transport per CS |
| DI/CS | - dissipation energy flux per CS |
| RC/CS | - % of active reaction centres per CS in comparison to the control |
| Plant growth parameters | |
| LMA | - leaf mass per area (g m^-2^) |
| FW | - fresh weight (g) |
| DW | - dry weight (g) |
